# Salt tolerance evaluation and key salt-tolerant traits at germination stage of upland cotton

**DOI:** 10.3389/fpls.2024.1489380

**Published:** 2025-01-23

**Authors:** Mengjie An, Xinhui Huang, Yilei Long, Yin Wang, Yanping Tan, Zhen Qin, Xiantao Ai, Yan Wang

**Affiliations:** ^1^ Xinjiang Key Laboratory of Biological Resources and Genetic Engineering, College of Life Science & Technology, Xinjiang University, Urumqi, Xinjiang, China; ^2^ College of Smart Agriculture (Research Institute), Xinjiang University, Urumqi, Xinjiang, China

**Keywords:** salinization, crop variety, membership function, ion content, evaluation model

## Abstract

Cotton is an important cash crop with a certain salt tolerance, but its germination stage is very susceptible to the damage of salt stress, causing significant yield loss. However, few studies have evaluated the cotton salt tolerance and selected salt tolerance traits at germination stage. Therefore, in this study, 16 cotton samples with geographical representation were randomly selected from 308 cotton germplasms to determine the optimal 200 mmol·L^-1^ NaCl in cotton germination experiments. On this basis, the salt tolerance of 308 upland cotton varieties and the growth, ion distribution and transport of highly salt-tolerant and non salt-tolerant cotton germplasms were analyzed. The results showed that the 308 germplasms were classified into five classes through cluster analysis, i.e, (1) highly salt-tolerant germplasms (HST, 49), (2) salt-tolerant germplasms (ST, 169), (3) moderately salt-tolerant class (MST,43), (4) lowly salt-tolerant germplasms (LST, 16), and (5) non-salt-tolerant germplasms (NST, 31). By calculating the salt tolerance index (STI) of various cotton germination and growth parameters and principal component analysis, combined with the correlation analysis and linear regression between mean membership function value (MFV) and STI, the key indexes of cotton germination and growth under salt stress, including total fresh weight, shoot fresh weight, and shoot length, were determined. In addition, three salt tolerance evaluation models constructed with different variables (6 variables in Model 1; 3 variables in Model 2; 1 variable in Model 3) found that the total fresh weight was the most reliable trait for the salt tolerance evaluation. In practical application, the variable selection for modelling could be adjusted based on the experimental workload. The comparison of the K^+^, Na^+^, and Ca^2+^ contents between HST and NST found that the higher the salt tolerance of cotton germplasms, the lower the Na^+^ content in the root system. Besides, the ion ratios and ion selective transport coefficients (ST) was found to be significantly positively correlated with the salt tolerance of cotton. This study will provide a basis for evaluating and breeding salt-tolerant cotton germplasms.

## 1 Introduction

Soil salinization in arid and semi-arid regions causes a quick decrease in the global arable land area (Peng et al., 2018). Especially, soil salinization causes salt stress to crops, exacerbating food shortages (Shelke et al., 2017). The impact degree of salt stress depends on the growth stage, variety, and salt tolerance of crops (Zhang et al., 2022). Slow growth is a mechanism of crops to reduce water consumption and salt absorption to adapt to salt stress (Munns and Millar, 2023). The initial and direct impacts of salt stress are inhibition of seed germination and hypocotyl growth (Wang et al., 2016). Some crops have evolved intrinsic tolerances to cope with salt stress. Previous studies have shown that salt-tolerant plants have less biomass reduction and better growth than non-salt-tolerant plants under salt stress (Ahmed et al., 2013). The reason for the difference may be the ion transfer and exchange in plants under salt stress. Salt-tolerant plants have the ability to maintain high K^+^/Na^+^ and Ca^2+^/Na^+^ ratios and reabsorb Na^+^ into vacuoles (Munawar et al., 2021). Besides, salt-tolerant plants can also segregate excess Na^+^ and Cl^-^ and accumulate compatible osmolytes under high salinity conditions (Asghar et al., 2023). Therefore, the best way to use salinized lands is to select, breed, and plant salt-tolerant crop varieties.

Cotton (*Gossypium hirsutum* L.), an important fiber crop in the world (Dong et al., 2023), has a certain salt tolerance. Some salt-tolerant cotton varieties can avoid excessive accumulation of Na^+^ by increasing Na^+^ efflux from roots and leaves or isolating Na^+^ in vacuoles, and cope with nutrient imbalance by regulating K^+^, Na^+^, and Ca^2+^ transport to maintain ion homeostasis (Wang et al., 2021). Therefore, cotton has been considered a pioneer crop for salinized soil remediation. However, the salt tolerance threshold of cotton is only 7.7 dS m^-1^ (Sharif et al., 2019). When the contents of Na^+^, Cl^-^, and other ions in the soil are too high, the biochemical, molecular, and physiological processes of cotton could still be damaged, leading to decreases in growth rate, photosynthetic rate, leaf and root size, biomass, yield, yield components, and fiber quality (Maryum et al., 2022; Guo et al., 2024). Studies have shown that cotton yield decreases by 15% when soil salinity is in the range of 8-10 dS m^-1^ and by 55% when soil salinity reaches 18 dS m^-1^ (Satir and Berberoglu, 2016; Zörb et al., 2019). However, there are differences in the responses of different crop germplasms to salt stress. According to the identification of salt tolerance in cotton seedlings, the Na^+^ content in the roots and stems of salt-tolerant cotton germplasms was high after salt stress, while the Na^+^ content in the upper and lower epidermal cells, palisade cells, and sponge parenchyma cells of non-salt tolerant cotton leaves was higher than that of salt-tolerant germplasms. At the same time, higher K^+^/Na^+^ ratios were observed in various parts of salt-tolerant cotton (Peng et al., 2016, 2023). In recent years, more and more research is focused on the selection and breeding of salt-tolerant germplasms. For example, Zhang et al. (2011) found that salt-tolerant cotton plants overexpressing the Arabidopsis gene *AVP1* had a 20% higher fiber yield than wild-type plants. Zafar et al. (2024) found that salt-tolerant cotton could survive under high salinity conditions and exhibited good fiber quality.

As is well known, salt stress has a prominent impact on the germination and seedling stages of cotton (Peng et al., 2023). Salt tolerance at germination stage plays a crucial role in the survival and growth of crops under salt stress in arid and semi-arid areas (Wu et al., 2019). However, most previous studies have focused on the seedling stage or late growth stage (Sikder et al., 2020; Sun et al., 2021; Peng et al., 2023). Identifying the germination-sage salt tolerance of cotton germplasms can provide assistance for salt-tolerant cotton breeding. There have been reports on the identification of germination-stage salt tolerance of 22 cotton germplasms, but the authors are more concerned with the germination rate and average germination time (Munawar et al., 2021). The germination of cotton germplasms is a qualitative change (Wu et al., 2019), and the growth of the embryonic root and embryo under salt stress varies depending on the germplasms’ salt tolerance. Therefore, using length and weight indicators to reflect quantitative changes can provide a more detailed and accurate evaluation of germplasms’ salt tolerance.

Different biological and environmental factors influence seed germination by different physiological and molecular mechanisms (Penfield, 2017). At present, many studies have focused on the evaluation of germination-stage salt tolerance of crops, but the evaluation methods, selected indexes, and mathematical models are different. Li et al. (2020) used analysis of variance and regression analysis to classify 552 sunflower germplasms into five salt tolerance levels, and identified germination index and germination potential index as the highest reliable traits. Tian et al. (2024) analyzed the morphological and physiological indexes of 41 maize varieties at germination and seedling stages using principal component analysis (PCA), subordinate function method (SFM), and GGE biplot, and identified salt-tolerant and non-salt-tolerant germplasms. In addition, some studies have used analysis of variance, PCA, correlation analysis, SFM, cluster analysis, and stepwise regression analysis jointly to evaluate the germination-stage salt tolerance of crops such as rapeseed, soybean, and pea (Wu et al., 2019; Khan et al., 2022; Guan et al., 2023). However, there are still few reports on the comprehensive evaluation of germination-stage salt tolerance of different cotton germplasms by multivariate statistical methods currently. Especially, the mathematical models in previous studies were all constructed with multiple indicators. However, in the breeding process, relying on measuring multiple germination indicators to determine salt tolerance is often time-consuming and laborious (Biermann et al., 2022). The models constructed with multiple indicators assign different weights to each variable feature, which can also easily ignore variables with low importance and miss information on key variables (Toda et al., 2021). Therefore, there is still a lack of progressive and simplified mathematical models for salt-tolerant germplasm selection at the cotton germination stage. To achieve efficient selection and breeding of salt-tolerant cotton varieties, it is important to develop an efficient and practical system for the evaluation of the germination-stage salt tolerance of cotton.

Therefore, to shorten the selection cycle of salt-tolerant cotton germplasms and increase the selection efficiency and accuracy, 308 cotton germplasms from different regions were selected for experiments, and a cotton germplasm salt tolerance evaluation system for germination stage was established. Most of the germplasms used in this study came from the northwest region of China, so arid and semi-arid regions could be used as target areas for salt-tolerant cotton. The objectives of this study were: (1) to determine the optimal salt concentration for salt-tolerant germplasm selection at the germination stage of cotton; (2) to identify cotton germplasms of different salt-tolerance levels and cotton varieties suitable for salt stress conditions; (3) to identify the most reliable growth traits for germination-stage salt tolerance evaluation; (4) to establish and verify a salt tolerance prediction model based on the identified reliable growth traits; and (5) to clarify the relationship between root/shoot ion content and salt tolerance of cotton under salt stress. This study will systematically elucidate the mechanism of the germination-stage salt tolerance in cotton and provide an important basis for the selection and breeding of salt-tolerant upland cotton varieties.

## 2 Materials and methods

### 2.1 Materials

In this constant-temperature incubator germination experiment conducted from April 2023 to April 2024, the laboratory temperature was controlled at 25°C. A total of 308 cotton germplasms were obtained from the China Germplasm Resource Bank, which came from different regions (Northwest China 30.7%, Yellow River basin of China 32.3%, Yangtze River basin of China 14.0%, Northeast China 14.3%, Other Countries 8.7%) (**Supplementary Figure S1**). The collected germplasms were preserved in the seed bank of Xinjiang Academy of Agricultural Sciences.

### 2.2 Determination of the optimal NaCl concentration for salt tolerance evaluation

Sixteen germplasms with geographical representation were randomly selected from the 308 germplasms for germination in NaCl solutions (0, 100, 150, 200 mmol·L^-1^), to determine the optimal salt concentration for salt-stressed germination experiments. Sterile water (0 mmol·L^-1^ NaCl) treated seeds were used as the control. Twelve healthy and intact seeds with black epidermis were randomly selected for each germplasm, sterilized with 75% ethanol for 1 min, soaked in 4% sodium hypochlorite solution for 8 min, and rinsed with sterile water for 5 times. The seeds were then wrapped with two sheets of filter paper, and placed in the solutions with different NaCl concentrations. The filter paper absorbing the solution was used as the substrate for seed germination. Thirty six seeds for each germplasm were in each concentration of NaCl solution. The hydroponic device for germination was kept in an constant-temperature incubator in complete darkness (temperature: 25 ± 1°C/25 ± 1°C (day/night); relative humidity: 65%). In the first 7 days, there was no need to replace the salt solution and filter paper. On the 7th day, normal growing seedlings were selected for measurements. The device was placed in the dark and constant-temperature incubator for the rest of the time to avoid the influence of external environment on the germination experiment. Seed was considered to have germinated when the radicle length was greater than half the seed length (Munawar et al., 2021; Xiao et al., 2021). The six germination indexes germination rate (GR), root length (RL), shoot length (SL), root fresh weight (RFW), shoot fresh weight (SFW), and total fresh weight (TFW) were measured 7 days after germination. After that, the salt tolerance index (STI) (Equation 1) and salt injury index (SII) (Equation 2) were calculated (Wu et al., 2019), and the SII of the six indexes and different NaCl concentrations were regressed. The NaCl concentrations were calculated when the SII of each index was 0.5, to determine the optimal NaCl concentration for the evaluation of salt tolerance of cotton germplasms.


(1) $$\begin{array}{c} \text{Salt tolerance index (STI)}\\ =\text{Germination index value of }\\ \text{NaCl treatment}/\text{Germination index value of the control} \end{array}$$

(2) Salt injury index(SII)=1−STI

### 2.3 Selection of salt-tolerant germplasms

The 308 germplasms were divided into batches for 200 mmol·L^-1^ NaCl stress-germination experiment (three replicates per germplasm, 12 seeds per replicate). In the experiment, two treatments including the optimal NaCl concentration (200 mmol·L^-1^) selected before and the control (0 mmol·L^-1^ of NaCl concentration, steamed sterile water) were designed. The number of germinated seeds was recorded daily in the first 7 days, and the germination rate (GR) was calculated on the 7th day (Equation 3) (Alvarado et al., 1987; Ruan et al., 2002; Wu et al., 2019). Cotton SFW, RFW, TFW (g), SL, and RL (cm) were measured 7 days after sowing. To ensure the reliability of the data, extreme data was eliminated, and the cotton shoots with similar growth status were used to measure the six germination indexes.


(3) $$\begin{array}{c} \text{Germination rate(GR)}\\ =\text{Number of seeds germinated on day }7/\text{Total number of seeds}\\ \times 100\% \end{array}$$

### 2.4 Evaluation of salt tolerance

The membership function value (MFV) (Equation 4) was used to evaluate the salt tolerance of cotton germplasms (Wu et al., 2019; Sun et al., 2021).


(4) $$\text{MFV}=\left(\text{X}-\text{Xmin}\right)/\left(\text{Xmax}-\text{Xmin}\right)\times 100\%$$

where X is the actual measured STI (salt tolerance index) of the germplasm, and Xmax and Xmin are the maximum and minimum STI observed in all germplasms, respectively. The average MFV of each cotton germplasm was the average of MFVs of the STIs of GR, SFW, RFW, TFW, SL, and RL.

### 2.5 Analysis of ionic characteristics

Based on the germination-stage salt tolerance of 308 germplasms determined before, the 308 germplasms were divided into five classes, namely highly salt-tolerant germplasms (HST), salt-tolerant germplasms (ST), moderately salt-tolerant germplasms (MST), lowly salt-tolerant germplasms (LST), and non-salt-tolerant germplasms (NST). To explore the differences in ion distribution and ion homeostasis between HST and NST under salt stress, 1-2 germplasms of HST and NST were selected for experiment (0 and 200 mmol·L^-1^ NaCl treatments). The ion content of the shoots and roots of each germplasm was detected after 7-day salt stress (three replicates per germplasm per treatment, and twelve seedlings per replicate).

Specifically, the seedlings after salt stress were divided into shoots and roots, dried to constant weight in an oven, crushed with a tissue breaker, ground with a mortar, and sent to the testing center of Xinjiang University for ion content detection. Inductively coupled plasma optical emission spectrometry (optima 8000, ICP-OES, PerkinElmer, United States) was used to determine the K^+^, Ca^2+^, and Na^+^ contents in the roots and shoots, followed by calculation of K^+^/Na^+^ and Ca^2+^/Na^+^ ratios. The coefficients of K^+^, Ca^2+^, and Na^+^ selective transport (ST) (ST_Na_, ST_K_, ST_Ca_, ST_K/Na_, and ST_Ca/Na_) of shoots and roots were calculated according to the method of Glenn and Brown (1998).


(5) $${\text{ST}}_{\text{Na}}=({\text{Shoot}}_{{\text{Na}}^{+}}\text{ content})/({\text{Root}}_{{\text{Na}}^{+}}\text{ content})$$

(6) $${\text{ST}}_{\text{K}}=({\text{Shoot}}_{{\text{K}}^{+}}\text{ content})/({\text{Root}}_{{\text{K}}^{+}}\text{ content})$$

(7) $${\text{ST}}_{\text{Ca}}=({\text{Shoot}}_{{\text{Ca}}^{2+}}\text{ content})/({\text{Root}}_{{\text{Ca}}^{2+}}\text{ content})$$

(8) $${\text{ST}}_{\text{K}/\text{Na}}=({\text{Shoot}}_{{\text{K}}^{+}/{\text{Na}}^{+}}\text{ ratio})/({\text{Root}}_{{\text{K}}^{+}/{\text{Na}}^{+}}\text{ ratio})$$

(9) $${\text{ST}}_{\text{Ca}/\text{Na}}=({\text{Shoot}}_{{\text{Ca}}^{2+}/{\text{Na}}^{+}}\text{ ratio})/({\text{Root}}_{{\text{Ca}}^{2+}/{\text{Na}}^{+}}\text{ ratio})$$

### 2.6 Data analysis

Data were processed in Microsoft Excel 2016. Since the STI value was less than 1, data processing (arcsine transformation) was required, and the transformed data with a relative standard deviation less than 0.25 was used for subsequent analysis.

SPSS software (version 27.0.1) was used to conduct the t-test and F-test to test the significance of differences between samples of different treatments (*p* < 0.01).

Before PCA analysis, the raw data was standardized so that the mean of each variable was 0 and the variance was 1. Then, the “FactoMineR” and “factoextra” packages of R software 4.3.3 (https://www.rproject.org/) were used for PCA. The “PerformanceAnalytics” package of R software 4.3.3 was used for correlation analysis, and the “gcookbook” package of R software 4.3.3 was used for univariate linear regression analysis.

Based on the average MFVs of GR, SFW, RFW, TFW, SL, and RL under 200 mmol L^-1^ NaCl stress, the salt tolerance of 308 cotton germplasms was clustered. Firstly, the data of STI for each index was normalized, and then a clustering tree was drawn based on the average distance and the number of clusters (k = 5). The “ComplexHeatmap” package of R software 4.3.3 was used for cluster analysis, and the “gghalves” package of R software 4.3.3 was used to plot cloud and rain map.

The cotton germination indexes were used as independent variables (X), and the MFV was used as the dependent variable (Y) to construct mathematical models for predicting the salt tolerance of cotton germplasms. The “car” package of R software 4.3.3 was used for multicollinearity analysis. Root mean square error (RMSE), mean absolute error (MAE), and significance test (Sigma) were used to evaluate the model performance (Pathania et al., 2023). The RMSE shows how close the regression line is to the points of measured data. The MAE shows the average difference between the predicted value and the measured value. The Sigma shows that whether the relationship between the model and the dependent variable is caused by accidental factors.

## 3 Results

### 3.1 The optimal salt concentration for salt tolerance evaluation

**Figure 1:**
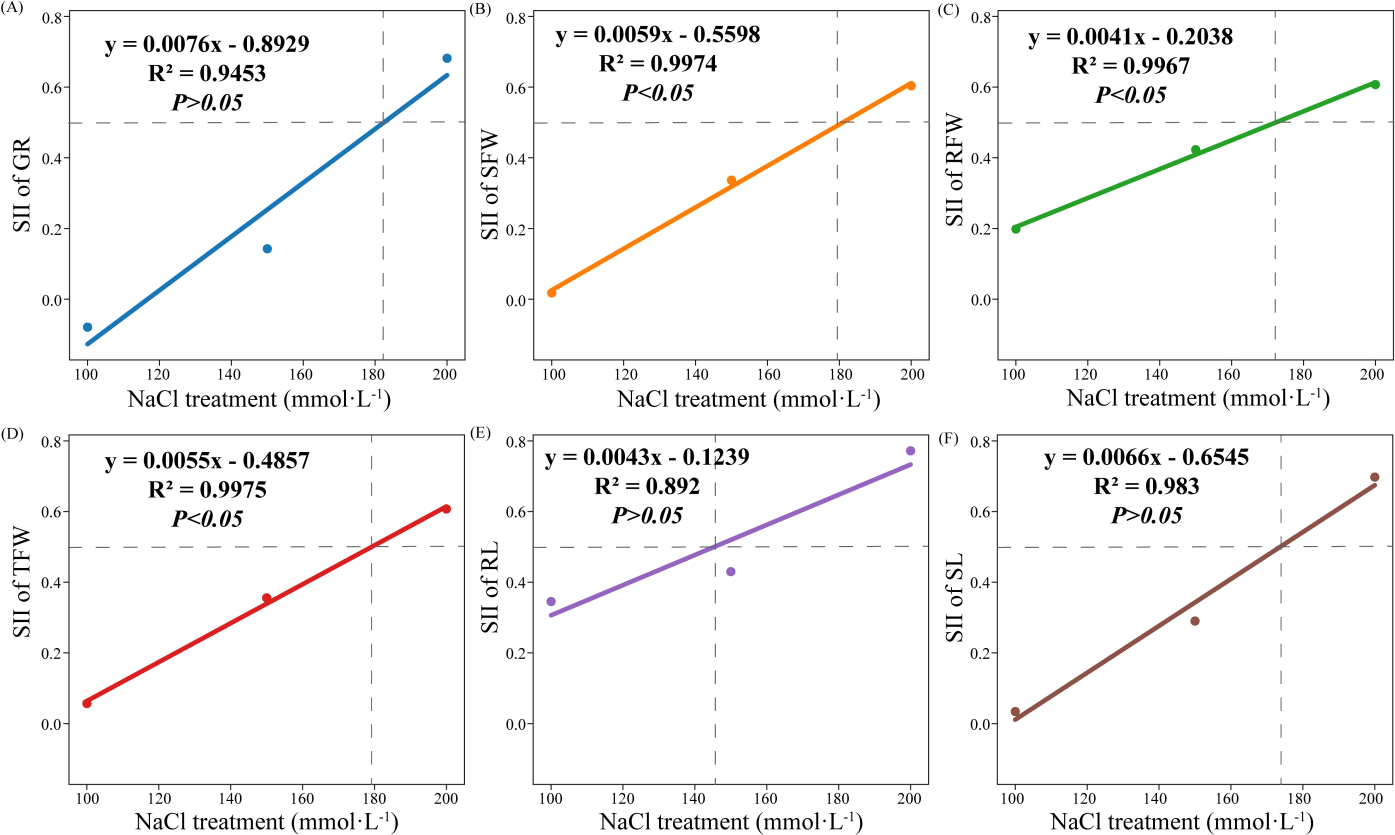
Selection of the optimal salt concentration for salt tolerance evaluation. The NaCl concentration of the SII was 0.5 of the GR **(A)**, SFW **(B)**, RFW **(C)**, TFW **(D)**, RL **(E)**, and SL **(F)** of 16 cotton germplasms under different NaCl concentrations. Data in the figure are means of 16 cotton germplasms for each indicator under each concentration of NaCl.

Linear regression analysis results showed that the NaCl concentration was 183.28 (**Figure 1A**), 179.63 (**Figure 1B**), 171.66 (**Figure 1C**), 179.22 (**Figure 1D**), 145.09 (**Figure 1E**), and 174.92 mmol·L^-1^ (**Figure 1F**) when the SII of GR, SFW, RFW, TFW, RL, and SL was 0.5, respectively. The NaCl concentrations were all greater than 170 mmol·L^-1^ when the SII of SFW, RFW, and TFW was 0.5. Besides, the linear regression equations established between NaCl concentrations and SFW, RFW, and TFW reached a significant level (*p* < 0.05). Therefore, 200 mmol·L^-1^ NaCl concentration was used to evaluate the salt tolerance of the 308 cotton varieties.

### 3.2 Genotypic variations in salt tolerance

**Figure 2:**
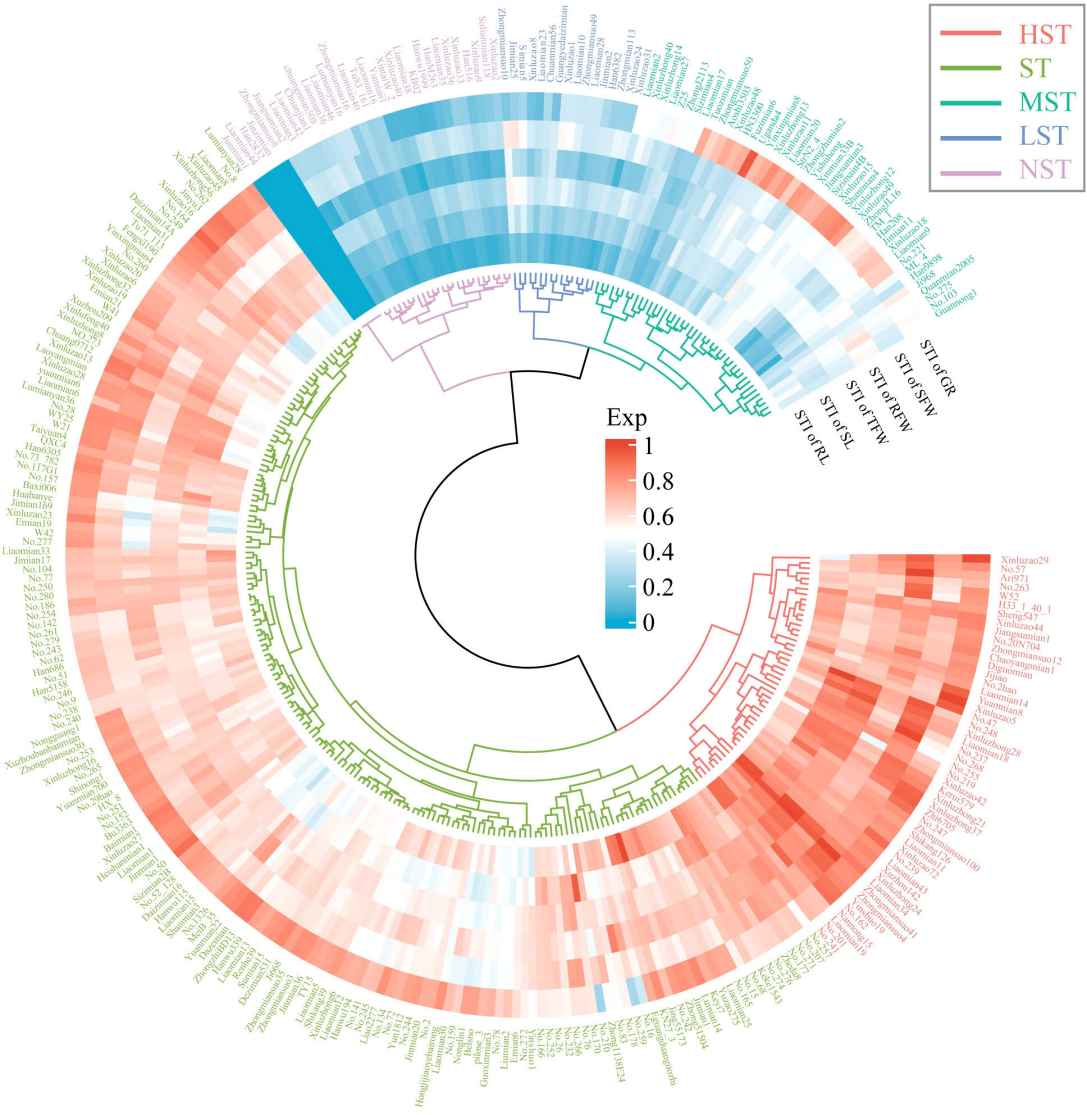
Cluster analysis of salt tolerance of 308 cotton varieties under NaCl stress. Different colors represent different salt-tolerant cotton varieties.

**Figure 3:**
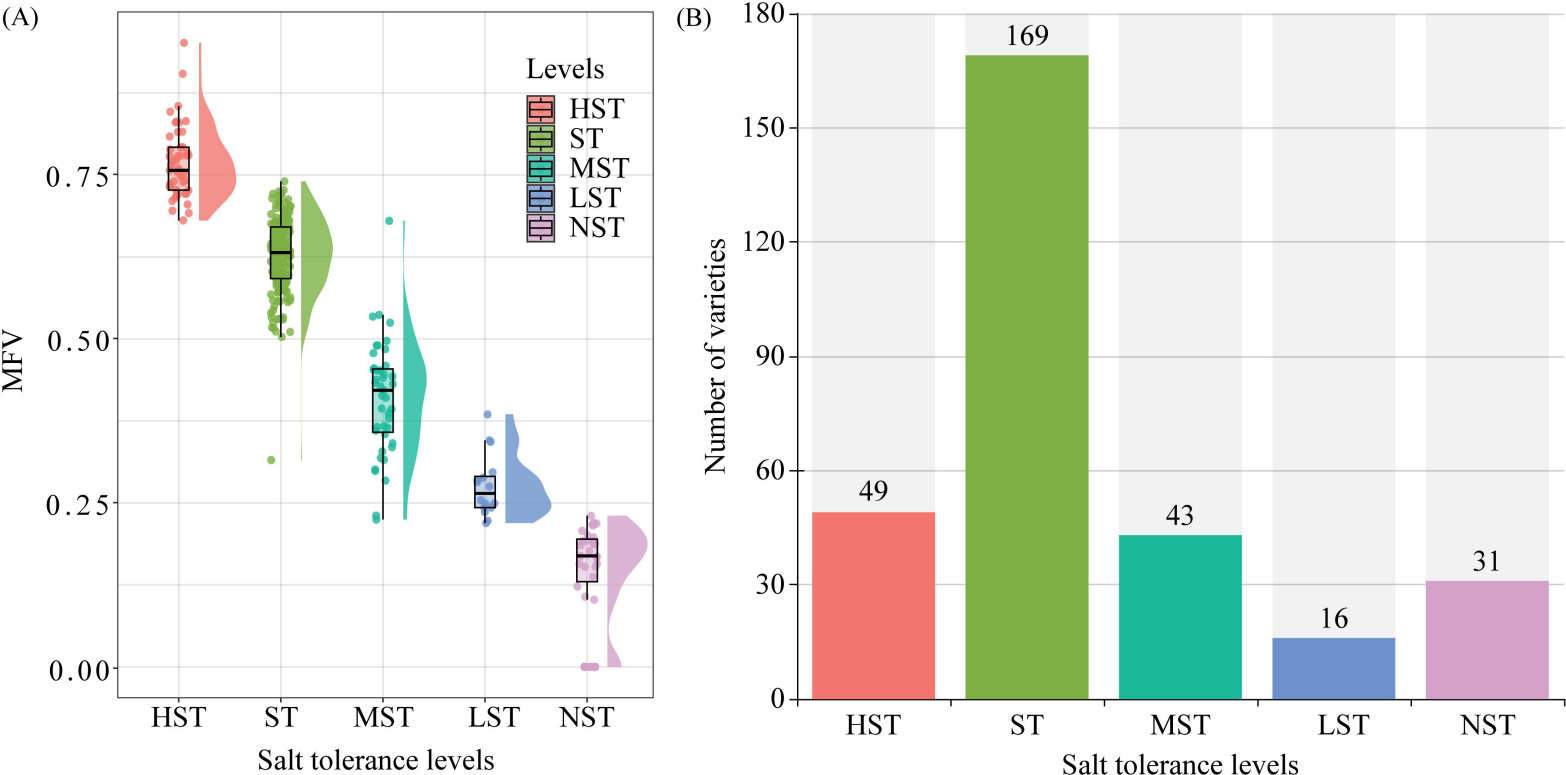
Membership function values (MFVs) for the classes with different salt tolerance. **(A)** Raincloud plot of MFVs distribution; **(B)** Number of different salt tolerances varieties.

Zhongmiansuo41, Liaomiansuo34, Zhongmiansuo4, Yinshuo19, and No.162 were the top 5 germplasms in HST (**Figure 2**). No.274, KN27_3, No.273, Zheda8 and WY25 were the top 5 germplasms in ST. Xinluzao13, Shanmian4, Xinluzao15, Xinluzhong12, and Jiangsumian3 were the top 5 germplasms in MST. Xinluzao8, Liaomian28, Zhongmian113, Xinluzao31 and Liaomian23 were the top 5 germplasms in LST. Jinzimian, Zhongmiansuo8, Han2832, Liaomian44, and Junmian1 were the top 5 germplasms in NST. The HST class had 49 germplasms, with MFVs in the range of 0.68 - 0.95 and an average MFV of 0.77 (± 0.06). The ST class had 169 germplasms, with MFVs in the range of 0.50 - 0.74 and an average MFV of 0.63 (± 0.06). The MST class had 43 germplasms, with MFVs in the range of 0.28 - 0.54 and an average MFV of 0.41 (± 0.09). The LST class had 16 germplasms, with MFVs in the range of 0.22 - 0.38 and an average MFV of 0.28 (± 0.05). The NST class had 31 germplasms, with MFVs in the range of 0.00 - 0.23 and an average MFV of 0.15 (± 0.07) (**Figures 3A, B**).

### 3.3 Determination of reliable salt-tolerant growth traits

**Figure 4:**
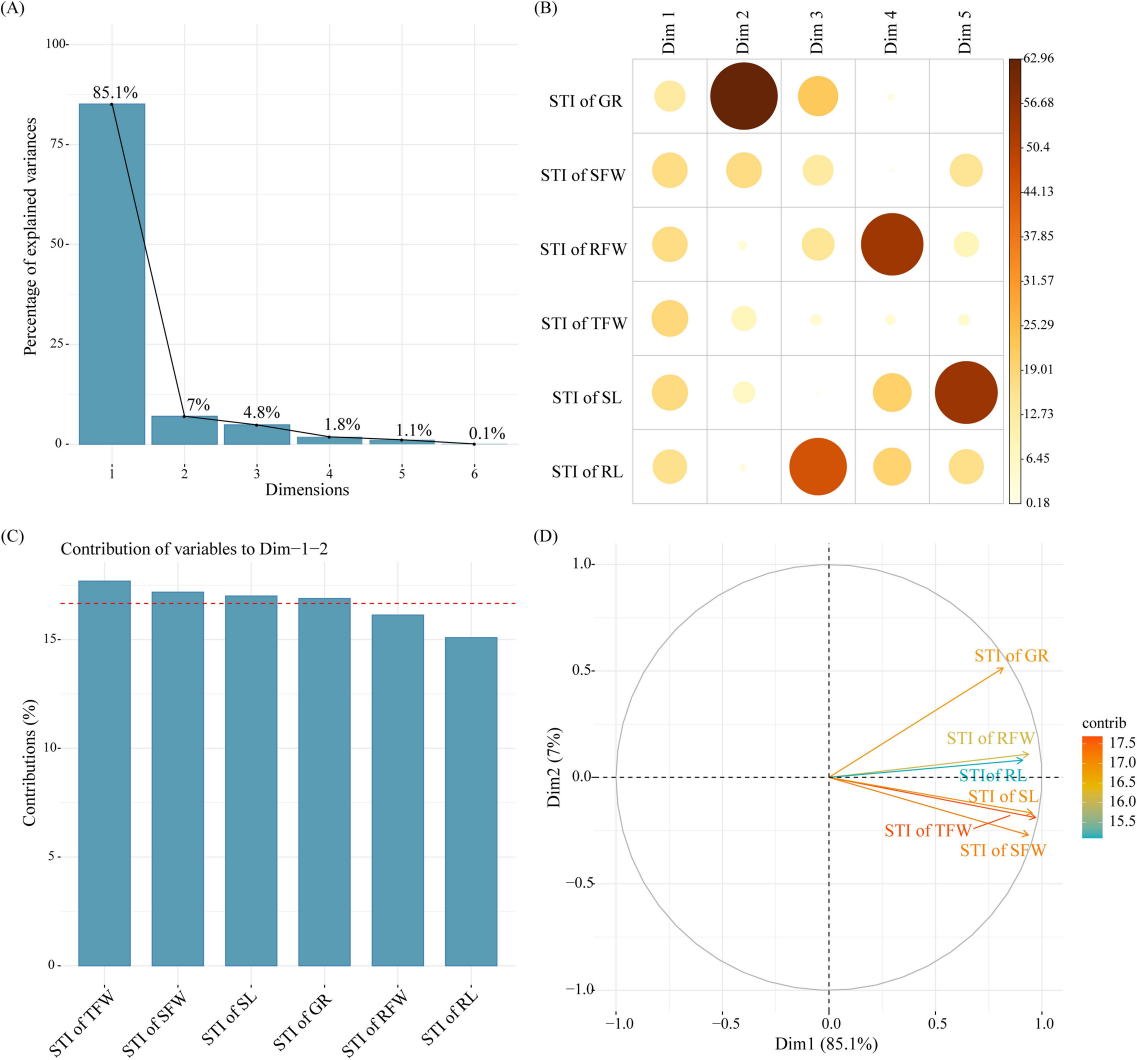
Principal component analysis (PCA) of cotton germination indexes. **(A)** Scree plot; **(B)** Correlation of the contributions of germination indexes to each Dim; **(C)** Comprehensive contribution of germination indexes to Dim1+Dim2; **(D)** PCA plot.

The proportions of the variance explained in the PCA of the six cotton germination indexes were as follows: Dim1 = 85.1%, Dim2 = 7.0%, Dim3 = 4.8%, Dim4 = 1.8%, Dim5 = 1.1%, and Dim6 = 0.1%. The cumulative proportion of the variance explained by Dim1 and Dim2 was 92.1% (**Figure 4A**). Therefore, Dim1 and Dim2 could represent most of the information of cotton germination indexes. All the indexes had high positive values in the principal components of Dim1. Therefore, Dim1 could represent the information of cotton germination growth. The STI of GR contributed the highest to the principal components of Dim2. Therefore, Dim2 could reflect the GR of cotton seeds (**Figure 4B**). At the same time, the total contribution (Dim1 + Dim2) of the six cotton germination indexes was as follows: STI of TFW > STI of SFW > STI of SL > STI of GR > STI of RFW > STI of RL (**Figure 4C**), and the STI of TFW, STI of SL, and STI of SFW were located in the same quadrant, indicating a strong correlation (**Figure 4D**). Therefore, the STI of TFW, STI of SFW, and STI of SL could be used as indicators to evaluate the germination-stage salt tolerance of cotton.

**Figure 5:**
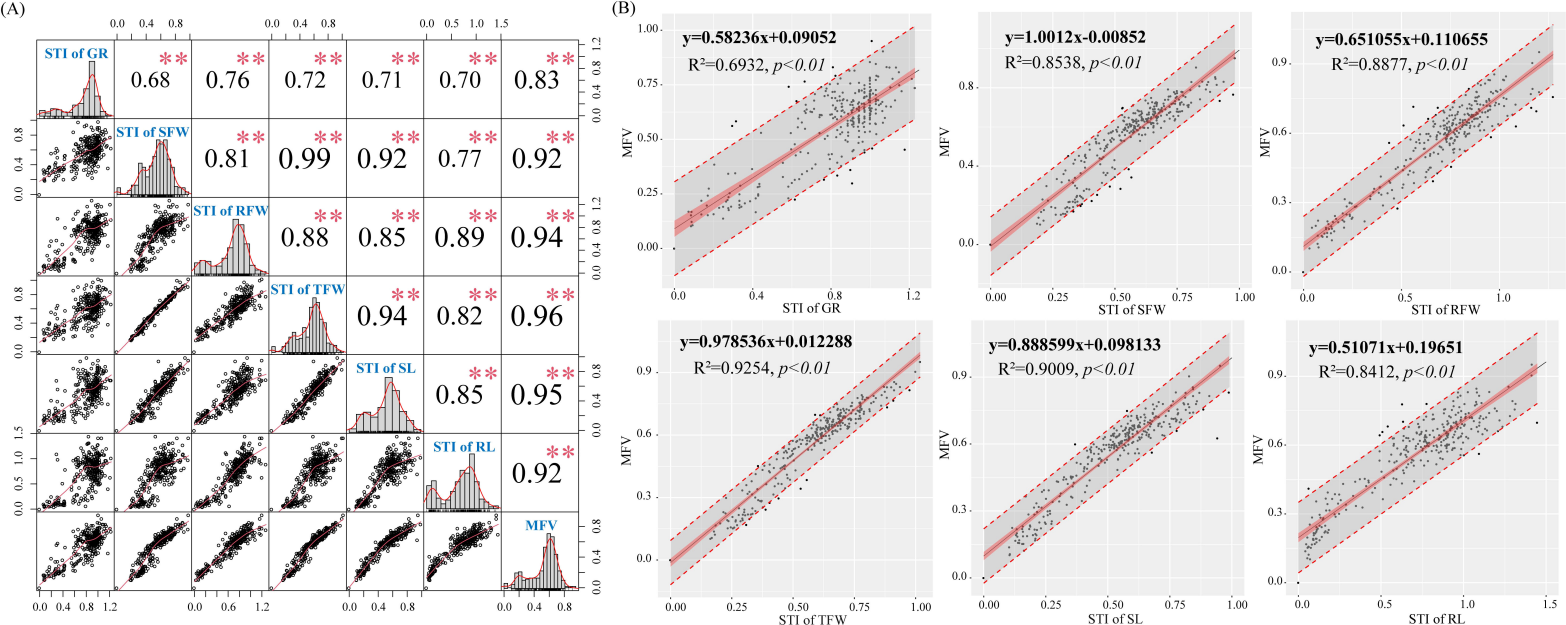
Relationship between membership function values (MFVs) and cotton germination indexes. **(A)** Correlation analysis of cotton germination indexes (n=308, **p<0.001, 95% confidence intervals); **(B)** Linear regression of the STI (salt tolerance index) of cotton germination indexes and average MFVs of different cotton varieties (n=308, *p<0.01*, 95% confidence intervals).

Correlation analysis (**Figure 5A**) showed that MFV was positively correlated with STI of GR (r = 0.83), STI of SFW (r = 0.92), STI of RFW (r = 0.94), STI of TFW (r = 0.96), STI of SL (r = 0.95), and STI of RL (r = 0.92) (*p* < 0.001). At the same time, the slope of the fitted equation and the correlation coefficient R^2^ can reflect the response of MFV to different germination indexes. From the fitted equations, it could be seen that the slopes of the fitted equations for STI of SFW, STI of TFW, and STI of SL were high (**Figure 5B**). When the STI of SFW, STI of TFW, and STI of SL increased by 0.1, the MFVs increased by 0.100, 0.098, and 0.089, respectively. The R^2^ of STI of TFW, STI of SL, STI of RFW, STI of SFW, and STI of RL were 0.93, 0.90, 0.89, 0.85, and 0.84, respectively. Therefore, the MFV was mainly affected by STI of TFW, STI of SFW, and STI of SL. Finally, TFW, SFW, and SL were selected as three key traits for the evaluation of cotton germination-stage salt tolerance.

### 3.4 Fitting of mathematical models

**Figure 6:**
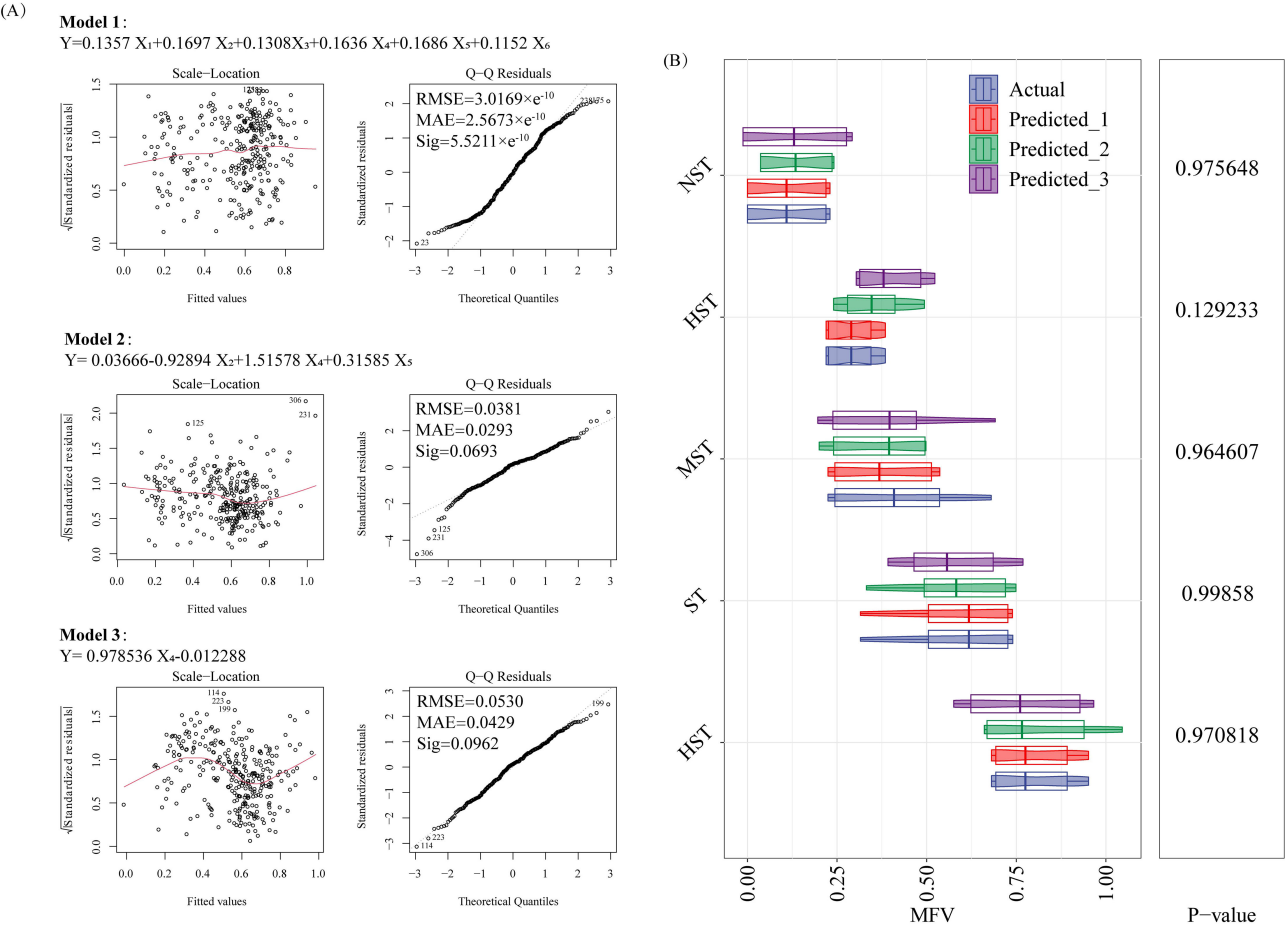
Construction **(A)** and validation **(B)** of mathematical models. Y, membership function value (MFV); X_1_, salt tolerance index (STI) of germination rate (GR); X_2_, STI of shoot fresh weight (SFW); X_3_, STI of root fresh weight (RFW); X_4_, STI of total fresh weight (TFW); X_5_, STI of shoot length (SL); X_6_, STI of root length (RL); Predicted_1, predicted value of Model 1; Predicted_2, predicted value of Model 2; Predicted_3, predicted value of Model 3.

According to the residual-fitting diagram of model evaluation, the points on both sides of the horizontal lines of Model 1, Model 2, and Model 3 are randomly distributed, indicating that the three models satisfy homoscedasticity. From the Q-Q diagram, it can be seen that the points of Model 1, Model 2, and Model 3 all roughly follow a straight line, indicating that the residuals of the three models approximately follow a normal distribution (**Figure 6A**). The equation of Model 1 was: MFV = 0.1357 STI of GR + 0.1697 STI of SFW + 0.1308 STI of RFW + 0.1636 STI of TFW + 0.1686 STI of SL + 0.1152 STI of RL; The RMSE was 3.0169×e^-10^, the MAE was 2.5673×e^-10^, and the Sigma was 5.5211×e^-10^. The equation for Model 2 was: MFV = 0.03666-0.92894 STI of SFW + 1.51578 STI of TFW + 0.31585 STI of SL; The RMSE was 0.0381, the MAE was 0.0293, and the Sigma was 0.0693. The equation for Model 3 was: MFV = 0.978536 STI of TFW-0.012288; The RMSE was 0.0530, the MAE was 0.0429, and the Sigma was 0.0962. All above indicated the reliability of the three model estimates. Based on this, **Figure 6B** shows the actual and model-predicted MFVs for different tolerance classes. There was no significant difference between the MFVs of Predicted_1, Predicted_2, Predicted_3 and the measured MFVs (*p* > 0.05), indicating that the MFV could be estimated by substituting the STI of each cotton germination index into the three mathematical models.

### 3.5 Analysis of ion characteristics of highly salt-tolerant and non-salt-tolerant germplasms

**Figure 7:**
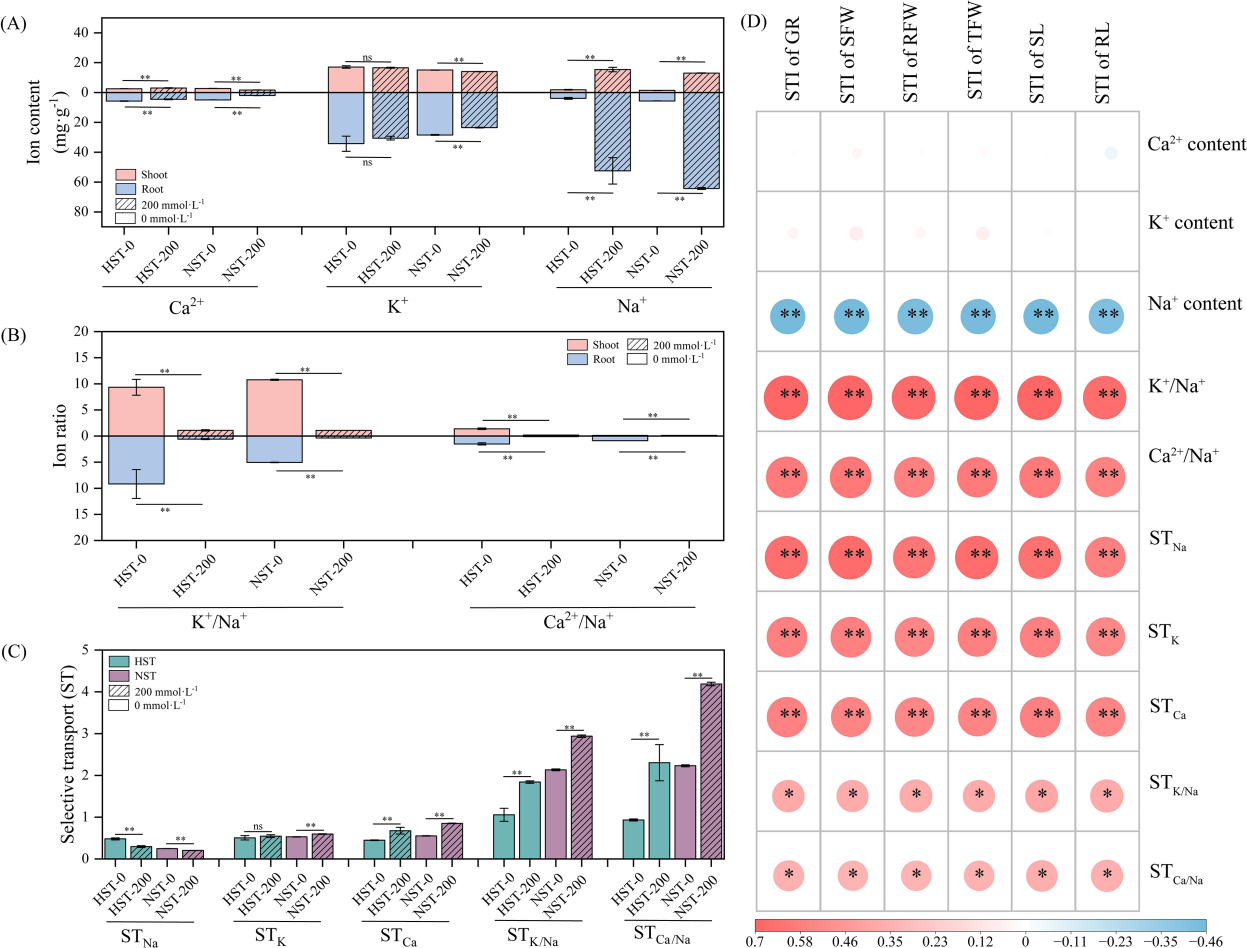
Analysis results of Na^+^, K^+^, and Ca^2+^ in the shoots and roots of germplasms with extreme salt tolerance levels [i.e., highly salt-tolerant germplasm (HST) and non-salt-tolerant germplasm (NST)] at germination stage under salt stress. **(A)** Ion contents; **(B)** Ion ratios; **(C)** Coefficients of ion-selective transport; **(D)** Correlation analysis between ion indexes and growth indexes (Pearson). t-test was used for pairwise comparison. *p < 0.05; **p < 0.01.

There were differences in ion content (**Figure 7A**), ion ratios (**Figure 7B**), and ion transport (**Figure 7C**) among cotton varieties with different salt tolerances. On the whole, before salt stress, the K^+^ content in plants was the highest, followed by Ca^2+^ content and Na^+^ content. After salt stress, the Na^+^ content in plants was the highest, followed by K^+^ content and Ca^2+^ content.

The shoot Ca^2+^ content, shoot Na^+^ content, root Na^+^ content, ST_Ca_, ST_K/Na_, and ST_Ca/Na_ of HST-200 increased by 18.73%, 729.13%, 1249.22%, 50.85%, 74.11%, and 147.05%, respectively (*p* < 0.01), compared with those of HST-0. The shoot K^+^/Na^+^ ratio, shoot Ca^2+^/Na^+^ ratio, root Ca^2+^ content, root K^+^/Na^+^ ratio, root Ca^2+^/Na^+^ ratio, and ST_Na_ of HST-200 decreased by 88.34%, 85.79%, 20.93%, 93.54%, 94.06%, and 38.30%, respectively (*p* < 0.01), compared with those of HST-0.

The shoot Na^+^ content, root Na^+^ content, ST_K_, ST_Ca_, ST_K/Na_, and ST_Ca/Na_ of NST-200 increased by 835.29%, 1040.75%, 12.82%, 53.97%, 37.61%, and 87.79%, respectively (*p* < 0.01), compared with those of NST-0. The shoot Ca^2+^ content, shoot K^+^ content, shoot K^+^/Na^+^ ratio, shoot Ca^2+^/Na^+^ ratio, root Ca^2+^ content, root K^+^ content, root K^+^/Na^+^ ratio, root Ca^2+^/Na^+^ ratio, and ST_Na_ of NST-200 decreased by 37.11%, 6.79%, 90.03%, 93.28%, 59.15%, 17.38%, 92.76%, 96.42%, and 18.01%, respectively (*p* < 0.01), compared with those of NST-0.

The correlation analysis (**Figure 7D**) and path analysis (**Supplementary Figure S2**) showed that the cotton germination indexes (STI of TFW, STI of SFW, STI of SL, STI of GR, STI of RFW, STI of RL) were negatively correlated with Na^+^ content in plants (*p* < 0.01), and positively correlated with K^+^/Na^+^ ratio (*p* < 0.01), ST_Ca_ (*p* < 0.01), and ST_Ca/Na_ in plants (*p* < 0.05).

## 4 Discussion

In the selection of the optimal salt concentration for the germplasm salt tolerance evaluation, the tested indexes must show obvious stress traits at the optimal concentration, while the plants grow normally (Zhou et al., 2023). Salt injury index (SII) represents the degree of salt stress-induced damages to plants, which is a concept opposite to the STI. The higher the SII, the severer the salt stress on plants. By calculating the SII, different germplasms can be compared in terms of the damage degree under the same salt stress, distinguishing between salt-tolerant and non-salt-tolerant germplasms (Wu et al., 2019). In the selection of the optimal salt concentration for the salt tolerance evaluation of rapeseed, soybean, wheat, eggplant, and mustard germplasms, the standard of damage approaching 50% in tested indexes under salt stress was also used (Wu et al., 2019; Choudhary et al., 2021; Zhou et al., 2023; Aggarwal et al., 2024; Gyanagoudar et al., 2024). However, at present, the optimal salt (NaCl) concentration for evaluating the germination-stage salt tolerance of cotton germplasms has not been definitively concluded. Some studies have shown that the germination rate of different cotton varieties under NaCl stress (150 mmol·L^-1^) shows a significant decrease compared with that of the control, thus 150 mmol·L^-1^ can be used as the optimal concentration for the selection of salt-tolerant cotton germplasms (Chen et al., 2021; Dong et al., 2023). However, some studies have also shown that 200 mmol·L^-1^ NaCl stress reduces the K^+^ content of cotton organs, root length, root surface area, and root volume, and increases the Na^+^ content of cotton organs, average root diameter, and root tissue density compared with the control, thus 200 mmol·L^-1^ can be used as the optimal concentration for the evaluation of salt tolerance of cotton germplasms (Sikder et al., 2020; Hou et al., 2023). The difference in the response of cotton to NaCl concentration at germination stage may be attributed to the genotype difference, i.e., different genotypes have different salt tolerance. It is worth noting that the selection of optimal NaCl concentration in most previous studies lack comparative analysis of multiple cotton varieties. In this study, the NaCl concentration gradient experiments with 16 cotton germplasms showed that the NaCl concentrations were all greater than 170 mmol·L^-1^ when the SIIs of GR, SFW, RFW, TFW, and SL were 0.5 (**Figure 1**). This shows that there is the greatest difference in germination indexes among different cotton varieties under 170 mmol·L^-1^ NaCl stress. Based on this, to ensure that cotton germplasms can exhibit salt stress-induced growth inhibitions and facilitate the differentiation of germplasms’ salt tolerance levels, the NaCl concentration 200 mmol·L^-1^ was used to select salt-tolerant germplasms from the 308 cotton germplasms.

However, salt tolerance in cotton is a complex quantitative trait, and a single indicator may not be sufficient to reflect the salt tolerance level of cotton plants (Quamruzzaman et al., 2022). Therefore, in this study, the MFV was used to comprehensively evaluate the salt tolerance of 308 cotton germplasms. Among them, the average MFV is a comprehensive indicator of salt-tolerant germination indexes (Aggarwal et al., 2024), and the STI reflects the salt tolerance potential of germplasms under salt stress (Wu et al., 2019). Selecting genotypes based on different growth indexes’ STI values and STI values-derived MFV is considered a reliable method for evaluating salt stress tolerance in *Brassica napus* (Wu et al., 2019), *Brassica juncea* (Aggarwal et al., 2024), *Morus alba* (Acharya et al., 2024), and wheat (Choudhary et al., 2021). When all indexes are positively correlated with salt tolerance, the membership function and average membership function are calculated for all growth indexes, and the salt tolerance of germplasms can be comprehensively considered based on multiple indexes (Wu et al., 2019). The stronger the tolerance of a germplasm to salt stress, the closer the growth indexes under salt stress are to those under control conditions, that is, the less negative impact of salt stress on the growth of the germplasm. Therefore, the larger the calculated STI and the closer the MFV of each index to 1, the mean MFV calculated based on the average of the MFVs of all indicators is larger. Therefore, the larger the mean MFV, the higher the germplasm’s salt tolerance (Wu et al., 2019). Studies have used the average MFV to classify the salt tolerance of wheat (Choudhary et al., 2021), eggplant (Gyanagoudar et al., 2024), and *Brassica napus* L (Wu et al., 2019). For example, Gyanagoudar et al. (2024) classified eggplant germplasms into five classes: very highly salt-tolerant germplasms (> 0.79), highly salt-tolerant germplasms (0.79 - 0.65), moderately salt-tolerant germplasms (0.64 - 0.21), lowly salt-tolerant germplasms (0.20 - 0.07), and non-salt-tolerant germplasms (< 0.07). Wu et al. (2019) detected 50 very highly salt-tolerant lines, 115 highly salt-tolerant lines, 71 moderately salt-tolerant lines, 202 lowly salt-tolerant lines, and 111 non-salt-tolerant lines at the germination stage of *Brassica napus* L. In this study, 308 cotton germplasms were divided into five classes (49 HST, 169 ST, 43 MST, 16 LST, and 31 NST) according to STI based on cluster analysis results. The MFVs of HST were greater than 0.68, the MFVs of ST were in the range of 0.50 - 0.74, the MFVs of MST were in the range of 0.28 - 0.54, the MFVs of LST were in the range of 0.22 - 0.38, and the MFVs of NST were smaller than 0.23. Based on this, the salt-tolerant cotton genotypes identified in this study are important resources and genetic materials for further breeding.

At present, there are many studies on the selection of cotton growth traits that indicate salt tolerance, and most of them focus on the differential responses of cotton physiological and molecular characteristics (Peng et al., 2023). However, no clear conclusion has been reached on the germination-stage salt tolerance indicators of cotton. Ayaz et al. (2000) reported that salt stress decreased the germination rate due to the dysregulation of seed metabolism. Seed germination is not a quantitative change but a qualitative change (Sun et al., 2021). Therefore, the evaluation of germination-stage salt tolerance of cotton germplasms cannot be based only on germination rate. In the PCA of cotton germination indexes, it was found that three traits (STI of TFW, STI of SFW, and STI of SL) had a significant effect on the germination of cotton germplasms. Combined with correlation analysis and linear regression results, that is, MFV was positively correlated with STI of TFW (r=0.96), STI of SFW (r=0.92), and STI of SL (r=0.95) (p<0.001). These indicate that the changes in MFV values were mainly influenced by the changes in STI of TFW, STI of SFW, and STI of SL during cotton seed germination. This is consistent with the conclusions of previous studies on the selection of salt tolerance traits in other plants. In the study of salt tolerance in *Solanum nigrum*, fresh weight (FW) was the growth index most relevant to plant salt tolerance, and the FW of stems and leaves significantly decreased as the salt concentration increased (Ortega-Albero et al., 2023). In the study of salt tolerance in *Morus alba*, the survival rate-related salt tolerance index was significantly positively correlated with root length, shoot length, and shoot weight (Acharya et al., 2024). In the evaluation of germination-stage salt tolerance of eggplant germplasms, correlation analysis and regression analysis between various indexes and the average membership function showed that germination rate and seedling length-related indexes were reliable traits for evaluation (Gyanagoudar et al., 2024). In the study of salt tolerance in wheat germplasms, SL was positively correlated with RL and SFW, and SFW was identified as the most reliable trait for evaluating germination-stage salt tolerance (Choudhary et al., 2021). In the study of salt tolerance of *Abelmoschus esculentus* germplasms, there was a strong correlation between salt stress and the decrease in germination rate and fresh dry weight of *A. esculentus* varieties, thus these two traits were serve as the basis for evaluating the salt tolerance potential (Ullah and Jan, 2024). Therefore, in this study, total fresh weight (STI of TFW), shoot fresh weight (STI of SFW), and shoot length (STI of SL) were identified as the main germination-stage traits for selecting salt-tolerant cotton germplasms. Based on this, the analysis of the growth characteristics of cotton germplasms during germination and the determination of these key indexes provided a benchmark evaluation for salt tolerance of different cotton varieties (Dong et al., 2020).

To evaluate the salt tolerance of cotton germplasms easily and accurately, three models were constructed by using multiple linear regression of MFV and STI, combined with the selected reliable salt-tolerant growth traits. Using the three models, the salt tolerance of cotton germplasms was predicted by calculating the Y value. According to the Y values, the 308 cotton germplasms were divided into different salt tolerance classes. The higher the Y value, the higher the salt tolerance (Gyanagoudar et al., 2024). It is worth noting that the three mathematical models for the evaluation of germination-stage salt tolerance of cotton germplasms could be used for the evaluation of salt tolerance of other cotton germplasms, without the need to include a large number of germplasms for comparison. However, the variables in these three models are different. The six variables in Model 1 have high complexity and accuracy. However, this may increase experimental time and cost, and lead to a decrease in model generalization ability (Nhu et al., 2019; Kardani et al., 2022). The three variables in Model 2 and one variable in Model 3 have low complexity and high generalization ability, but may have low prediction accuracy and cannot capture subtle features of the data well (Nhu et al., 2019; Kardani et al., 2022). When measuring multiple traits is difficult and time-consuming, based on the average MFV and the RMSE, MAE, Sigma of the three models, the TFW of STI can be used as a single reliable trait for the evaluation of germination-stage salt tolerance of cotton germplasms. This is consistent with previous studies on the response of plant fresh weight to salt stress (Sikder et al., 2020; Birhanie et al., 2022). Research has shown that salt stress significantly affects growth by reducing plant stem length, leaf number, and leaf area, decreasing photosynthetic rate and ultimately total biomass (Sikder et al., 2020). Besides, in the study of salt stress on the growth and physiological changes of *Hibiscus cannabinus*, Birhanie et al. (2022) found that NaCl stress significantly reduced plant height, leaf dry weight, and fresh weight compared with the control, and these growth indexes were significantly negatively correlated with the contents of hydrogen peroxide (H_2_O_2_), superoxide anion (O_2_^-^), and malondialdehyde (MDA). In the later research, appropriate mathematical models can be selected for analysis according to the workload. The constructed mathematical models in this study help to shorten the selection time while giving accurate classification results of tolerance levels.

Plant salt tolerance is closely related to the balance of salt-based ions in plants (Liang et al., 2024). Salt stress promotes the competition for Na^+^, K^+^, and Ca^2+^ in plants (Ran et al., 2022). When excess Na^+^ enters plant cells, it directly disrupts plant cell metabolism, triggers K^+^ and Ca^2+^ outflow, and interferes with the absorption of K^+^ and Ca^2+^ by plants. This leads to the over accumulation of Na^+^ in plants, inhibiting plant growth (Lu et al., 2023). Therefore, re-establishing the ion homeostasis in plants under salt stress is an important salt-tolerant strategy (Long et al., 2019). Consistent with previous study (Guo et al., 2019), this study found that a large amount of Na^+^ were accumulated in the shoots and roots of HST and NST under salt stress. Besides, under 200 mmol·L^-1^ NaCl stress, the Na^+^ content in the roots of cotton was higher than that in the shoots at germination stage. This indicates that both HST and NST accumulated Na^+^ in the roots at the early stage of salt stress (Ishikawa et al., 2022). It was also found that more K^+^ and Ca^2+^ were accumulated in the shoots and roots of HST, and the Na^+^ content in the roots was reduced, compared with those of NST. This indicates that HST have a higher Na^+^ efflux than NST (Tester and Davenport, 2003), which helps HST to maintain ion and osmotic homeostasis under salt stress (Bose et al., 2014). This also proves that HST tolerate salt stress by storing K^+^ and Ca^2+^ (Wang et al., 2022). Besides, the ion homeostasis maintaining ability of highly salt-tolerant germplasms may also be closely related to its ion transporters or signaling pathways. Research has shown that the well-defined salt-overly-sensitive (SOS) signaling pathway in Arabidopsis can export Na^+^ to external culture media or ectoplasts, where SOS1 controls the homeostasis of essential ions such as K^+^ and Ca^2+^, achieving salt tolerance. Overexpression or co-overexpression of *SOS1* with other salt-tolerant genes can significantly enhance salt tolerance in various plants (Balasubramaniam et al., 2023). The expression levels of *GhSOS1*, *GhSOS2*, and *GhCBL10* (SOS3) encoding SOS pathway proteins in the leaf plasma membrane-associated ion transporters and *GhNHX1* and *GhCAX1* encoding vacuolar membrane-associated ion transporters of Na^+^ and Ca^2+^ transporters of salt-tolerant cotton genotypes were significantly up-regulated after stress, compared with those of the salt-sensitive genotypes (Peng et al., 2016).

Previous studies have shown that K^+^ and Ca^2+^ have a certain antagonistic effect on Na^+^, and excess Na^+^ in plants will lead to relative deficiency of K^+^ and Ca^2+^, so K^+^/Na^+^ and Ca^2+^/Na^+^ ratios in cotton tissues are still considered to be positively correlated with salt tolerance (Dong et al., 2020; Yuan et al., 2023). This is consistent with the results of correlation analysis between K^+^/Na^+^ and Ca^2+^/Na^+^ ratios and germination indexes (STI of TFW, STI of SFW, STI of SL, STI of GR, STI of RFW, STI of RL) of HST and NST in this study. This indicates that the salt tolerance levels divided by MFV are closely related to K^+^, Ca^2+^, and Na^+^ equilibrium. The results further verify the reliability of the selected key salt-tolerant growth traits and mathematical models in this study. To further explore the dynamic changes in ion balance of the germplasms with different salt tolerance, this study evaluated the absorption and distribution of K^+^, Ca^2+^, and Na^+^ in various parts of plants by calculating ion selective transport coefficients (ST_Na_, ST_K_, ST_Ca,_ ST_K/Na_, and ST_Ca/Na_), and clarified the ion balance in cotton seeds under salt stress conditions (Wang et al., 2021). It was found that HST could transport more Na^+^ from the roots to the shoots compared with NST. This indicates that HST respond faster to Na^+^ over-accumulation at germination stage (Bose et al., 2015), which helps to maintain the ion homeostasis and protect the root system (Yuan et al., 2019). Finally, HST have better growth status/traits under salt stress. By analyzing the distribution and ratios of Na^+^, K^+^, and Ca^2+^, this study provides insights into the germination-stage salt tolerance mechanism of different cotton germplasms. In future research, the effects of salt stress on the growth and physiological characteristics of selected salt-tolerant genotypes at different growth stages (seedling, flowering, and bolling stages) will be further studied, which will help to more accurately select and widely apply salt-tolerant cotton genotypes (Sikder et al., 2020).

## 5 Conclusion

In the germination experiment with 308 cotton varieties, Zhongmiansuo41, Liaomiansuo34, Zhongmiansuo4, Yinshuo19, and No.162 were the top 5 varieties with high salt tolerance. These highly salt-tolerant cotton varieties can be used as genetic materials for further breeding. Three germination indexes (total fresh weight, shoot length, and shoot fresh weight) were determined to be the key indexes for evaluating the salt tolerance of cotton varieties by combining PCA, correlation analysis, and linear regression. The three prediction models constructed based on stepwise regression (6 variables in Model 1; 3 variables in Model 2; 1 variable in Model 3) could accurately predict the germination-stage salt tolerance of cotton. In addition, ion analysis results showed that Na^+^ content is a key factor affecting germination-stage salt tolerance. Highly salt-tolerant germplasms (HST) had higher K^+^ and Ca^2+^ contents and lower root Na^+^ content than non-salt-tolerant germplasms (NST). Therefore, HST maintains ion homeostasis by increasing root Na^+^ efflux and increasing the storage of K^+^ and Ca^2+^ in the plant, adapting to NaCl stress. These findings will help to rapidly evaluate the germination-stage salt tolerance, breed the salt-tolerant cotton varieties, and provide a basis for molecular marker-assisted selection of salt-tolerant germplasms.

However, temperature or nutrient transport during different growth stages have different effects on the salt tolerance of cotton. This study only evaluated the salt tolerance during the germination stage of cotton, and did not evaluate the salt tolerance during the seedling stage and the entire growth period. Therefore, further research is needed to determine whether the salt-tolerant germplasms selected in this study are equally salt-tolerant in other growth stages and whether the selected evaluation indexes are also applicable to the evaluation of salt tolerance in other growth stages. In addition, this study was only conducted indoors, and field experiments are needed in the later stage to combine the phenotypic characteristics and ion transport mechanisms of indoor and field experiments, to better select cotton germplasms suitable for planting in salinized areas.

## Data Availability

The original contributions presented in the study are included in the article/**Supplementary Material**. Further inquiries can be directed to the corresponding authors.
