# Supplementary material for: Salt tolerance evaluation and key salt-tolerant traits at germination stage of upland cotton
Source: Front Plant Sci. 2025 Jan 23;15:1489380. doi: 10.3389/fpls.2024.1489380 (PMC11799247; doi:10.3389/fpls.2024.1489380)
Supplement: Supplementary file 1 [file Table1.doc]

**Fig. S1. The origin of the 308 germplasms from different regions.**


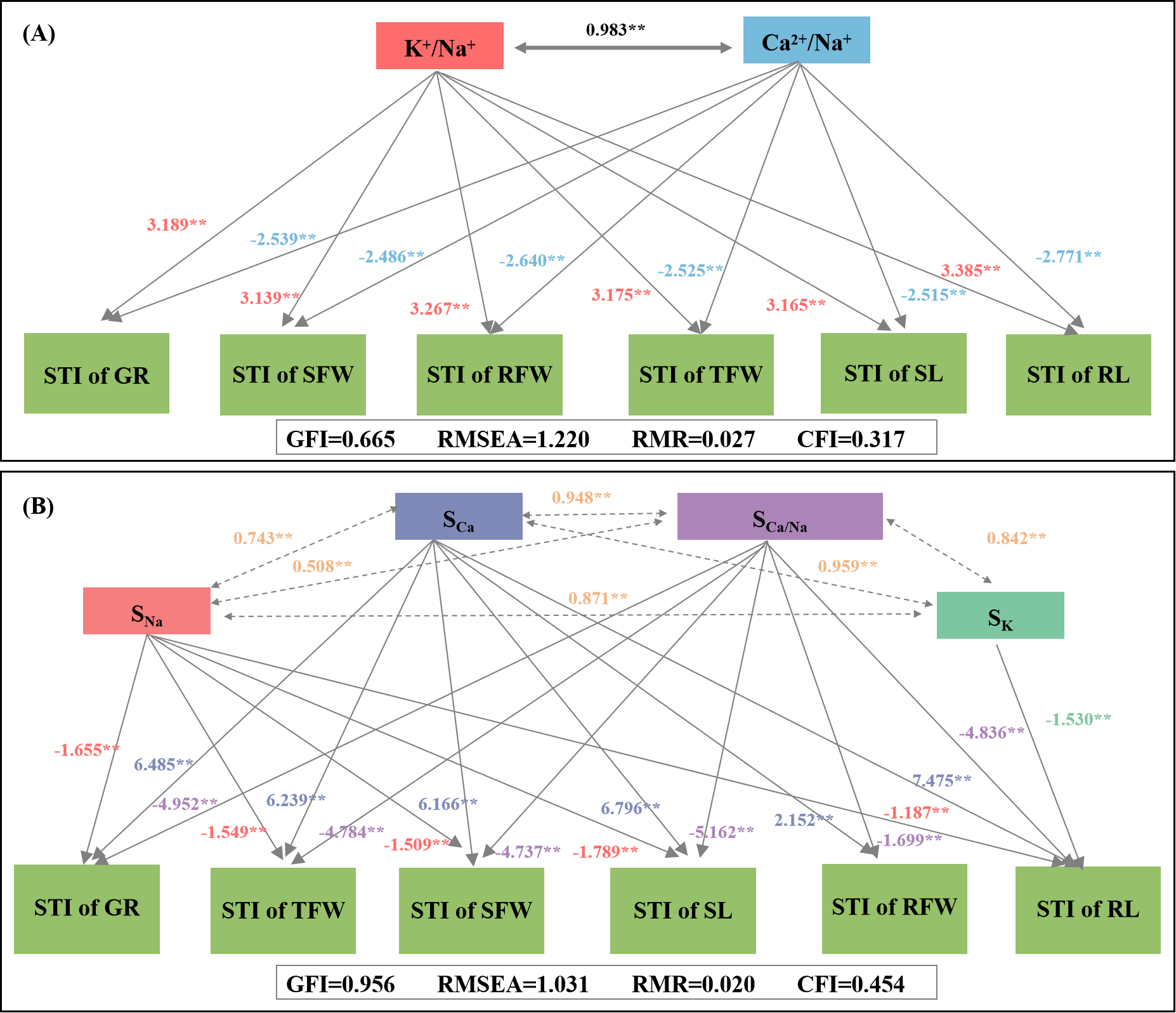


**Fig. S2. Path analysis between ion indexes and growth indexes. *, *p < 0.05*; **, *p < 0.01*.**
